# Complete Genome Sequence of Lactobacillus harbinensis Strain NSMJ42, Isolated from Makgeolli, a Traditional Korean Alcoholic Beverage

**DOI:** 10.1128/MRA.01177-19

**Published:** 2019-11-27

**Authors:** Ji Young Jung, Sang-Soo Han, Z-Hun Kim, Byung-Gon Ryu, Hyun Mi Jin, Eu Jin Chung

**Affiliations:** a Microbial Research Department, Nakdonggang National Institute of Biological Resources (NNIBR), Sangju-si, Gyeongsangbuk-do, South Korea; Loyola University Chicago

## Abstract

In the present work, we report the complete genome sequence of Lactobacillus harbinensis NSMJ42, isolated from makgeolli (a Korean traditional alcoholic beverage) in South Korea. The final genome assembly consists of a 3.29-Mbp chromosome with 3,082 protein-coding sequences and a G+C content of 53.36%.

## ANNOUNCEMENT

Makgeolli is a Korean traditional fermented alcoholic beverage with a 6 to 8% alcohol content that is brewed with rice and nuruk. Nuruk, a starchy disk or tablet formed from various cereals as raw material, contains diverse fungal and bacterial strains from the surrounding environment and acts as a starter culture for saccharification and alcoholic fermentation for producing makgeolli (1). Studies on the makgeolli microflora have revealed the presence of amylolytic molds (Aspergillus, Rhizopus, and Mucor spp.), alcohol-producing yeasts (Saccharomyces spp.), and lactic acid bacteria (LAB) in makgeolli (1–6). LAB are involved in the production of organic acids, amino acids, vitamins, and aromatic compounds during makgeolli fermentation and also in the prevention of bacterial contamination and spoilage by Micrococcus, Bacillus, Aerobacter, and Pseudomonas spp. (1, 7–9). It has been reported that makgeolli has medicinal properties like antioxidant, antihypertensive, antidiabetes, and anticancer activities (1, 10, 11). Moreover, probiotic properties of LAB and yeast isolates in makgeolli have been proven (12, 13). We isolated *Lactobacillus harbinensis* NSMJ42 from makgeolli and sequenced the whole genome to understand its whole metabolic capacity and functional potential.

A traditional makgeolli collected in Gyeongsangbuk Province (South Korea) was diluted in phosphate-buffered saline (PBS) (pH 7.4), and the dilutions were spread over an MRS agar (Difco) plate. The plates were incubated at 30°C for 48 h, and we obtained a single colony of strain NSMJ42. For whole-genome sequencing, genomic DNA was isolated from strain NSMJ42 grown in MRS broth (Difco) at 30°C, using a TruSeq DNA PCR-free kit (Illumina). The whole genome was sequenced at Cosmo Genetech (Seoul, South Korea) by a combination of the PacBio RS II single-molecule, real-time (SMRT) sequencing platform using a 20-kb SMRTbell template library and the Illumina NovaSeq 6000 platform (2 × 101 bp) with an insert size of 550 bp. A total of 70,372 postfilter polymerase reads (783,148,504 bp; mean read length, 11,128 bp) were generated from SMRT sequencing, and 100,364 subreads of clean data (781,769,715 bp; mean subread length, 7,789 bp) were produced with quality filtering (minimum polymerase read quality, 0.75; minimum polymerase read length, 50) and adapter trimming using HGAP.3 within PacBio’s SMRT Analysis v2.3.0 (14). To generate long and accurate sequences, preassembly was performed by mapping shorter subreads onto longer subreads (14,557-bp threshold) using HGAP.3 (14). The error-corrected 7,726 long subreads (84,586,769 bp; mean read length, 10,948 bp) were *de novo* assembled to the initial draft genome assembly by HGAP.3 (14). Additionally, 5,037.99 Gbp (1,531.01-fold coverage) with 49,881,092 paired-end reads were generated from the Illumina NovaSeq 6000 system. The raw Illumina reads were used for consensus genome polishing and error correction by mapping onto the initial PacBio draft genome assembly with HGAP.3 (14), and the resulting contig was circularized using NUCmer v3.1 and MUMmerplot v3.5 (15).

The final genome assembly, which had a mean coverage of 162.31-fold and a G+C content of 53.36%, consisted of a 3,290,626-bp circular chromosome. Average nucleotide identity (ANI) analysis was conducted with OrthoANIu (16) to the accurate identification of strain NSMJ42 and resulted in 97.97% similarity to *L. harbinensis* DSM 16991^T^ (GenBank accession number AUEH00000000). The value is higher than the ANI threshold of 95 to 96% (17), indicating that strain NSMJ42 belongs to the same species, *L. harbinensis*. The NSMJ42 genome was annotated on NCBI PGAP version 4.8 (18), and it contains 3,082 protein-coding genes, 15 rRNA genes, 67 tRNA genes, 4 noncoding RNAs, and 56 pseudogenes. BASys genome annotation (19) showed that specific clusters of orthologous groups (COGs) were assigned to 2,062 coding sequences (CDSs), and genes for carbohydrate transport and metabolism (G) showed the highest prevalence (10.4%), followed by genes for replication, recombination, and repair (L) (6.6%) and transcription (K) (6.1%). The strain NSMJ42 genome contains 160 carbohydrate-active enzyme (CAZyme) genes, as predicted by HMMER searches (E value, <1E−15; coverage, >0.35) in dbCAN (20), including 108 genes encoding glycoside hydrolases (GHs), 18 genes encoding carbohydrate esterases (CEs), 28 genes encoding glycosyltransferases (GTs), 2 genes encoding polysaccharide lyases (PLs), and 4 genes encoding carbohydrate-binding modules (CBMs) involved in the degradation or modification of carbohydrates and their subsequent utilization in fermentative metabolism. In addition, several cell surface proteins (class A and C sortases), LPXTG motif cell wall anchor domain proteins, and d-alanyl-lipoteichoic acid biosynthesis proteins (dltABCD) were detected in the strain NSMJ42 genome, which explains the potential of *L. harbinensis* NSMJ42 to adhere to the intestinal epithelial cells (21, 22). The bacteriocin genome-mining tool BAGEL4 (23) identified one area of interest (AOI) corresponding to class II bacteriocin.

### Data availability.

The genome sequence and raw sequencing reads for strain NSMJ42 were deposited under GenBank accession number CP041364, BioProject accession number PRJNA552757, BioSample accession number SAMN12217290, and SRA accession numbers SRX6406718 and SRX6406719.
